# Higher insoluble fiber intake is associated with a lower risk of prostate cancer: results from the PLCO cohort

**DOI:** 10.1186/s12889-024-17768-8

**Published:** 2024-01-19

**Authors:** Yang Shen, Qinbo Yuan, Minhong Shi, Banxin Luo

**Affiliations:** 1 Department of Urology, The Second Affiliated Hospital of Nanjing, University of Chinese Medicine, Nanjing, 210000 China; 2 Department of Urology, Affiliated Wuxi Fifth Hospital of Jiangnan University, Wuxi, 214000 China; 3 Department of Medical Prevention, Nantong Center for Disease Control and Prevention of Jiangsu Province, Nantong, 226007 China; 4 Nanjing Drum Tower Hospital Clinical College of Traditional Chinese and Western Medicine, Nanjing University of Chinese Medicine, 321 Zhongshan Road, Nanjing, 210000 China

**Keywords:** Dietary fiber, Insoluble fiber, Prostate cancer, PLCO

## Abstract

Studies regarding the relationship between fiber intake and prostate cancer (PCa) have conflicting results. Therefore, this study examined the relationship between fiber intake and the risk of PCa by using data from Prostate, Lung, Colorectal, and Ovarian (PLCO) Cancer Screening Trial. A total of 54,336 participants in the United States, consisting of 6,414 patients with PCa, were included in this study. Multivariate Cox regression models were applied to estimate adjusted hazard ratios (aHRs) and corresponding 95% confidence intervals (CIs). Compared with individuals in the lowest quartile, individuals in the highest quartile of insoluble fiber intake had a significantly lower risk of PCa (aHR, 0.87; 95% CI, 0.78–0.98). By contrast, no significant associations were detected between total fiber intake (aHR, 0.90; 95% CI, 0.80–1.01) or soluble fiber intake (aHR, 0.90; 95% CI, 0.80–1.02). Subgroup analyses showed that insoluble fiber was related to a decreased risk of PCa in subjects with the following characteristics: age > 65 years, nonsmoking or former smokers, education level ≤ high school, non-Hispanic white ethnicity, or without a family history of PCa. In addition, significant combined effects of insoluble fiber intake, age and family history of PCa on the risk of PCa were observed, but no combined effects of smoking status and insoluble fiber intake were observed. In addition, total fiber, insoluble fiber, and soluble fiber intake had no influence on the mortality of PCa patients. These results show that all 3 measures of fiber suggest a protective association, but insoluble fiber may have a stronger association with the risk of PCa. Future studies are warranted to further investigate these relationships.

**Supplementary Information:**

The online version contains supplementary material available at 10.1186/s12889-024-17768-8.

## Introduction

Prostate cancer (PCa) is one of the most common cancers in older men and has the second-highest incidence of all cancers in males worldwide [1]. Growing amounts of data indicate that dietary patterns are an influential risk factor for PCa [2]. There are significant differences in incidence among different geographic and ethnic populations, with Western European and Northern European countries being the most affected [3]. These findings may be closely related to differences in dietary intake habits [4]. A prospective cohort study of 217,937 men in the UK revealed a lower risk of PCa among vegetarian men than among nonwhole vegetarians [5]. In contrast to nonwhole vegetarian diets, consumption of vegetarian diets seem to protect against prostate cancer, which suggests that dietary intervention can be an effective strategy for PCa prevention [6–8].

Dietary fiber, such as nondigestible carbohydrates and the complex polymer, lignin, plays a critical role in our daily diet, is abundant in plants and has important biological features [9]. Based on its physical and chemical properties, dietary fiber can be divided into insoluble and soluble types [10, 11]. Insoluble dietary fiber is found mainly in bran and whole grain breads and cereals, and soluble dietary fiber is often found in grains such as oats and barley, legumes, and most fruits and vegetables [12, 13]. Soluble fibers are beneficial for reducing serum lipid levels, and insoluble fibers can promote laxation [14]. Many studies have revealed that dietary fiber protects against the development of cardiovascular disease [15], diabetes [16], and even cancer [17].

The association between fiber intake and prostate cancer risk has long been examined in many cohort and case-control studies in different populations [18–21]. Deschasaux et al. revealed an inverse association between dietary fiber intake and PCa risk in their 12.6-year follow-up study [22]. In addition, Sawada et al. reported that insoluble dietary fiber was associated with decreased PCa risk [23]. However, another study indicated that dietary fiber intake had no significant association with PCa risk [24]. Given the inconsistent epidemiological evidence on the associations between fiber intake and PCa risk, the present study aimed to investigate the relationship between dietary fiber intake and the risk and prognosis of PCa using data from the Prostate, Lung, Colorectal, Ovarian (PLCO) Cancer Screening Trial. We intended to perform a more systematic analysis to evaluate the factors associated with the effect of daily dietary fiber intake on PCa risk.

## Materials and methods

### Study population

The design of the PLCO trial has been described online and additional methods can be found on the following website: https://cdas.cancer.gov/learn/plco [25]. Between November 1993 and July 2001, nearly 155,000 participants aged 55–74 years were registered at ten clinical centers throughout the U.S. Individuals were randomly allocated to the intervention arm or the control arm. Participants were excluded if they did not respond to the baseline questionnaire (BQ), dietary history questionnaire (DHQ) or dietary questionnaire (DQX) at baseline. Participants in the control arm were offered standard treatment, while those in the intervention arm were invited to undergo PCa screening tests. Informed consent was obtained from all participants. This research was approved by the institutional review boards of all ten participating centers and the U.S. National Cancer Institute.

### Data collection

All participants were required to complete the BQ, which included information on age, race, weight, height, education, alcohol consumption, smoking, family history of PCa and other lifestyle variables. Then, two food-frequency questionnaires (DHQ and DQX) were used to collect dietary information. Participants in the intervention arm who were randomized before December 1995 were given the DHQ in 1999, and those who were randomly assigned at or after that time were given the DHQ generally around their third anniversary of randomization (T3). Patients in the control arm who were randomized before December 1998 were offered the DHQ in 1999 or 2000, and those who were randomized at or after that time were offered the DHQ at baseline. However, only those in the intervention arm responded to the DQX around the time they were randomized at baseline (T0). The nutrient variables used were based on values from the USDA’s 1994–1996 Continuing Survey of Food Intakes by Individuals (CSFII) and the University of Minnesota’s Nutrition Data Systems for Research. Nutrient intake was calculated by multiplying food frequencies and nutrient amounts in the database and summing all foods to obtain a total daily value for each nutrient.

### Assessment of PCa

The men in the intervention group underwent an annual blood draw for prostate-specific antigen (PSA) examination and a digital rectal examination (DRE) to detect PCa. If PCa was suspected at the time of screening, PCa diagnostic procedures were performed at that time. The PLCO trial confirmed the diagnosis of PCa through medical record abstraction (MRA) of the men by the following criteria: (1) a self-report of PCa on an annual study update; (2) an abnormal suspicious PSA level (> 4 ng/mL) or DRE screening; (3) a death certificate indicating PCa; (4) despite no indication of PCa during the trial, the Death Review Committee suspected PCa based on other indicators; or (5) a relative informed the screening center of the participant’s PCa diagnosis.

### Statistical analysis

Continuous variables are presented as the mean ± standard deviation (SD), and between-group differences were assessed by Student’s *t* test. Categorical data are presented as percentages, and the chi-square (*χ*^*2*^) test was used to compare the differences in categorical characteristics. Cox regression analysis was performed to calculate adjusted hazard ratios (aHRs) and 95% confidence intervals (95% CIs) for the risk and prognosis of PCa in relation to fiber intake. The multivariate Cox regression model was adjusted for age, body mass index (BMI), education, race, marital status, pack-years of smoking, alcohol consumption, total energy intake, total vegetable intake, total fruit intake, total calcium intake, total folate intake, family history of PCa, arm allocation and study center. In addition to the above covariates, the PSA exam results and the Gleason score were adjusted to evaluate the associations between fiber intake and PCa prognosis. All the statistical analyses were conducted with R 4.1.2. A two-sided *P* < 0.05 was considered to indicate statistical significance.

## Results

### Characteristics of the study participants

The characteristics of the participants are summarized in Table 1. A total of 54,336 men were recruited, including 6,414 PCa patients. The average age of participants in the PCa and control groups was 63.4±5.1 and 62.5±5.3, respectively. Approximately 73.5% of the patients in the PCa group had a BMI > 25, and 75.4% of the men in the control group had a BMI > 25. There were significant differences in the number of pack-years smoked and education levels between the PCa patients and cancer-free controls (*P* = 1.44E-13). However, alcohol consumption was not significantly different (*P* = 0.255). Most of the men were white, and the race distributions were markedly different between the PCa and control groups (*P* = 2.66E-16). Notably, significant differences were observed in marital status, family history and total fruit intake between the two groups. Additionally, total energy, vegetable, calcium and folate intake did not significantly differ between the PCa group and cancer-free group (*P* > 0.05).

**Table 1:** Baseline characteristics of study subjects in PLCO cohort

Characteristics	Controls			Cases		*P* ^*^
	No. of controls	%		No. of cases	%	
Total	47,922	88.20		6,414	11.80	
Age						3.47E-55
≤ 59 years	16,125	33.65		1,534	23.92	
60–64 years	15,005	31.31		2,210	34.46	
65–69 years	10,907	22.76		1,789	27.89	
≥ 70 years	5,885	12.28		881	13.74	
BMI (kg/m^**2**^)						5.18E-08
≤ 18.5	131	0.27		12	0.19	
> 18.5 and ≤ 25	11,649	24.31		1,690	26.35	
> 25 and ≤ 30	23,713	49.48		3,260	50.83	
> 30	10,817	22.57		1,242	19.36	
Missing	1,612	3.36		210	3.27	
Education						0.033
≤High school	17,925	37.40		2,319	36.16	
≥Some college	28,945	60.40		3,973	61.94	
Missing	1,052	2.20		122	1.90	
Race						2.66E-16
White, Non-Hispanic	42,676	89.05		5,781	90.13	
Black, Non-Hispanic	1,213	2.53		247	3.85	
Hispanic	817	1.70		92	1.43	
Asian	1,897	3.96		150	2.34	
Pacific Islander	250	0.52		23	0.36	
American Indian	99	0.21		10	0.16	
Missing	970	2.02		111	1.73	
Pack-year smoking						1.44E-13
Never	17,334	36.17		2,619	40.83	
≤ 20	9,485	19.79		1,273	19.85	
> 20	19,498	40.69		2,340	36.48	
Missing	1,605	3.35		182	2.84	
Drinking intensity						0.255
Never	11,284	23.55		1,496	23.32	
≤ 5	15,617	32.59		2,026	31.59	
> 5 and ≤ 10	5,468	11.41		777	12.11	
> 10 and ≤ 20	5,466	11.41		732	11.41	
> 20 and ≤ 30	4,425	9.23		631	9.84	
> 30	5,662	11.82		752	11.72	
Marital status						2.23E-07
Married	39,881	83.22		5,524	86.12	
Widowed	1,522	3.18		192	2.99	
Divorced	3,602	7.52		379	5.91	
Separated	396	0.83		47	0.73	
Never married	1,469	3.07		150	2.34	
Missing	1,052	2.20		122	1.90	
Family history						2.93E-31
No	42,567	88.83		5,471	85.30	
Yes	3,267	6.82		702	10.94	
Possibly-relative	720	1.50		89	1.39	
Missing	1,368	2.85		152	2.37	
Total energy intake (kcal/day)						0.062^†^
Mean ± SD	1,996 ± 814			1,975 ± 806		
Total vegetable intake (g/day)						0.929^†^
Mean ± SD	290 ± 194			291 ± 189		
Total fruit intake (g/day)						0.012^†^
Mean ± SD	265 ± 223			273 ± 222		
Total calcium intake (mg/day)						0.169^†^
Mean ± SD	922 ± 501			931 ± 498		
Total folate intake (mcg/day)						0.311^†^
Mean ± SD	609 ± 277			613 ± 277		

^*^ From chi-square test

^†^ From Student’s *t* test

### Associations between fiber intake and PCa risk

The median follow-up times for the PCa and control groups were 5.9 years and 11.5 years, respectively. Multivariate Cox regression analysis was applied to assess the associations between fiber intake and the risk of PCa. As shown in Table 2, compared with those of subjects in the lowest quartile of insoluble fiber (Q1), the aHRs of PCa risk were 0.97 (95% CI, 0.90–1.05; *P* = 0.453), 0.97 (95% CI, 0.90–1.06; *P* = 0.526), and 0.87 (95% CI, 0.78–0.98; *P* = 0.016) for groups Q2, Q3, and Q4, respectively. In addition, according to our quartile analyses, total fiber intake (Q4 vs. Q1: aHR, 0.90; 95% CI, 0.80–1.01; *P* = 0.073) and soluble fiber intake (Q4 vs. Q1: aHR, 0.90; 95% CI, 0.80–1.02; *P* = 0.086) were slightly lower but not significantly associated with the risk of PCa.

**Table 2:** Association between fiber intake and the risk of PCa using Cox regression analysis

Nutrients	Controls	Cases	aHR^*^	95% CI	*P*
Total fiber (g/day)					
Q1 (0.74–12.84)	12,006	1,584	1.00 (reference)		
Q2 (12.85–17.70)	11,992	1,607	0.96	0.89–1.04	0.346
Q3 (17.71–23.74)	11,934	1,638	0.97	0.89–1.05	0.420
Q4 (23.75–97.82)	11,990	1,585	0.90	0.80–1.01	0.073
Insoluble fiber (g/day)					
Q1 (0.42–8.34)	12,028	1,578	1.00 (reference)		
Q2 (8.35–11.60)	11,986	1,617	0.97	0.90–1.05	0.453
Q3 (11.61–15.69)	11,890	1,660	0.97	0.90–1.06	0.526
Q4 (15.70–65.67)	12,018	1,559	0.87	0.78–0.98	0.016
Soluble fiber (g/day)					
Q1 (0.31–4.31)	12,053	1,589	1.00 (reference)		
Q2 (4.32–5.90)	11,983	1,615	0.98	0.91–1.06	0.638
Q3 (5.91–7.91)	11,915	1,643	0.98	0.89–1.06	0.576
Q4 (7.92–36.66)	11,971	1,567	0.90	0.80–1.02	0.086

^*^Multivariate Cox regression model was adjusted for entry age, BMI, pack year smoking, alcohol drinking intensity, total energy, total vegetable intake, total fruit intake, total calcium intake, total folate intake, education, race, marital status, study center, arm and family history

### Subgroup analyses for the effect of fiber intake on PCa risk

We applied subgroup analyses to evaluate the effect of insoluble fiber intake on PCa risk stratified by age, BMI, smoking status, drinking status, education level, race and family history. The results of these analyses are shown in Table 3. Compared with participants in the lowest quartile of insoluble fiber intake (Q1), there was a significantly lower risk of PCa in the group with the highest quartile of insoluble fiber intake (Q4) among men with aged > 65 years (aHR, 0.72; 95% CI, 0.60–0.87; *P* = 0.001), nonsmokers (aHR, 0.79; 95% CI, 0.67–0.94; *P* = 0.007), former smokers (aHR, 0.85; 95% CI, 0.72–0.99; *P* = 0.038), those with an education level ≤ high school (aHR, 0.83; 95% CI, 0.69–1.00; *P* = 0.047), non-Hispanic whites (aHR, 0.86; 95% CI, 0.77–0.96; *P* = 0.010) and those without a family history of PCa (aHR, 0.87; 95% CI, 0.77–0.98; *P* = 0.020). However, a positive association was observed among current smokers (aHR, 1.49; 95% CI, 1.03–2.16; *P* = 0.033). Notably, that in the subgroup analysis stratified by age, total fiber intake and soluble fiber intake were associated with a 30% and 29%, respectively, decreased risk of PCa among men aged > 65 years. In addition, similar results were obtained among smokers (for total fiber intake: aHR, 0.79; 95% CI, 0.66–0.95; *P* = 0.010; for soluble fiber intake: aHR, 0.79; 95% CI, 0.65–0.95; *P* = 0.013).

**Table 3:** Subgroup analysis of the associations between fiber intake and PCa risk based on selected covariates

Variables		Total fiber				Insoluble fiber				Soluble fiber		
Age		aHR^*^	95% CI	*P*		aHR^*^	95% CI	*P*		aHR^*^	95% CI	*P*
Age ≤ 65	Q1	1.00 (reference)				1.00 (reference)				1.00 (reference)		
	Q2	1.01	0.92–1.11	0.818		1.02	0.93–1.12	0.674		1.00	0.91–1.10	0.962
	Q3	1.07	0.96–1.19	0.241		1.09	0.98–1.20	0.127		1.03	0.92–1.15	0.598
	Q4	1.02	0.88–1.18	0.806		0.99	0.86–1.13	0.847		0.96	0.82–1.11	0.555
Age > 65	Q1	1.00 (reference)				1.00 (reference)				1.00 (reference)		
	Q2	0.89	0.79–1.01	0.078		0.91	0.80–1.03	0.133		0.99	0.88–1.13	0.920
	Q3	0.80	0.70–0.93	0.002		0.82	0.71–0.94	0.004		0.87	0.75–1.00	0.055
	Q4	0.70	0.57–0.84	< 0.001		0.72	0.60–0.87	0.001		0.81	0.66–0.99	0.042
BMI												
BMI ≤ 25	Q1	1.00 (reference)				1.00 (reference)				1.00 (reference)		
	Q2	1.05	0.91–1.22	0.500		1.04	0.90–1.20	0.579		1.06	0.92–1.23	0.408
	Q3	1.10	0.94–1.29	0.250		1.09	0.93–1.27	0.313		1.00	0.85–1.18	0.957
	Q4	0.88	0.71–1.09	0.226		0.88	0.72–1.09	0.244		0.89	0.71–1.11	0.291
BMI > 25	Q1	1.00 (reference)				1.00 (reference)				1.00 (reference)		
	Q2	0.95	0.87–1.04	0.292		0.96	0.88–1.05	0.354		0.95	0.87–1.04	0.305
	Q3	0.92	0.83–1.02	0.103		0.92	0.84–1.02	0.109		0.97	0.87–1.07	0.518
	Q4	0.91	0.79–1.04	0.164		0.89	0.78–1.01	0.070		0.92	0.79–1.06	0.246
Smoking												
No	Q1	1.00 (reference)				1.00 (reference)				1.00 (reference)		
	Q2	0.94	0.84–1.06	0.330		0.93	0.83–1.04	0.186		0.91	0.81–1.03	0.131
	Q3	0.96	0.84–1.09	0.523		0.91	0.80–1.04	0.165		0.92	0.81–1.06	0.243
	Q4	0.79	0.66–0.95	0.010		0.79	0.67–0.94	0.007		0.79	0.65–0.95	0.013
Yes, currently	Q1	1.00 (reference)				1.00 (reference)				1.00 (reference)		
	Q2	0.94	0.72–1.22	0.633		1.06	0.81–1.39	0.654		0.93	0.72–1.21	0.605
	Q3	1.08	0.81–1.45	0.589		1.17	0.88–1.57	0.290		0.94	0.70–1.27	0.694
	Q4	1.20	0.82–1.76	0.359		1.49	1.03–2.16	0.033		1.22	0.82–1.82	0.316
Yes, former	Q1	1.00 (reference)				1.00 (reference)				1.00 (reference)		
	Q2	1.00	0.90–1.12	0.956		0.99	0.89–1.10	0.794		1.01	0.91–1.12	0.865
	Q3	0.96	0.85–1.08	0.512		0.96	0.85–1.08	0.507		1.00	0.88–1.13	0.976
	Q4	0.93	0.79–1.09	0.378		0.85	0.72–0.99	0.038		0.96	0.80–1.14	0.603
Drinking												
No	Q1	1.00 (reference)				1.00 (reference)				1.00 (reference)		
	Q2	0.94	0.71–1.23	0.635		0.93	0.71–1.21	0.582		0.96	0.72–1.26	0.742
	Q3	0.85	0.62–1.16	0.307		0.76	0.56–1.04	0.083		0.89	0.65–1.23	0.484
	Q4	0.75	0.48–1.16	0.191		0.70	0.46–1.07	0.098		0.64	0.40–1.01	0.057
Yes, currently	Q1	1.00 (reference)				1.00 (reference)				1.00 (reference)		
	Q2	0.96	0.88–1.05	0.338		0.97	0.89–1.05	0.427		0.97	0.89–1.06	0.500
	Q3	0.99	0.90–1.09	0.836		0.99	0.90–1.09	0.817		0.97	0.88–1.07	0.537
	Q4	0.94	0.82–1.07	0.328		0.91	0.80–1.03	0.138		0.96	0.84–1.10	0.561
Yes, former	Q1	1.00 (reference)				1.00 (reference)				1.00 (reference)		
	Q2	1.05	0.86–1.27	0.662		1.10	0.90–1.34	0.369		1.09	0.90–1.34	0.378
	Q3	0.96	0.77–1.20	0.732		1.05	0.84–1.31	0.647		1.05	0.83–1.32	0.700
	Q4	0.78	0.57–1.06	0.113		0.84	0.63–1.13	0.258		0.80	0.58–1.11	0.179
Education												
≤high school	Q1	1.00 (reference)				1.00 (reference)				1.00 (reference)		
	Q2	0.92	0.81–1.04	0.175		0.91	0.81–1.03	0.151		0.99	0.88–1.12	0.900
	Q3	0.94	0.82–1.08	0.361		0.98	0.85–1.12	0.710		0.93	0.80–1.07	0.308
	Q4	0.79	0.66–0.96	0.018		0.83	0.69–1.00	0.047		0.88	0.72–1.08	0.229
≥some college	Q1	1.00 (reference)				1.00 (reference)				1.00 (reference)		
	Q2	1.00	0.91–1.11	0.933		0.99	0.90–1.08	0.778		1.00	0.91–1.11	0.938
	Q3	1.01	0.90–1.12	0.908		0.97	0.88–1.08	0.631		1.01	0.90–1.12	0.925
	Q4	0.96	0.83–1.11	0.572		0.90	0.78–1.03	0.124		0.92	0.79–1.08	0.304
Race												
White, non–hispanic	Q1	1.00 (reference)				1.00 (reference)				1.00 (reference)		
	Q2	0.98	0.91–1.06	0.668		0.96	0.89–1.04	0.303		0.97	0.90–1.05	0.469
	Q3	0.97	0.89–1.06	0.523		0.96	0.88–1.05	0.364		0.96	0.88–1.05	0.355
	Q4	0.91	0.81–1.03	0.137		0.86	0.77–0.96	0.010		0.88	0.77–1.00	0.042
Other	Q1	1.00 (reference)				1.00 (reference)				1.00 (reference)		
	Q2	1.09	0.84–1.43	0.521		1.09	0.83–1.43	0.528		1.08	0.82–1.40	0.594
	Q3	1.19	0.89–1.59	0.247		1.27	0.95–1.69	0.104		1.11	0.83–1.49	0.475
	Q4	1.00	0.67–1.50	0.993		0.98	0.66–1.46	0.930		1.06	0.70–1.60	0.772
Family history												
Yes	Q1	1.00 (reference)				1.00 (reference)				1.00 (reference)		
	Q2	0.86	0.69–1.08	0.203		0.91	0.73–1.15	0.437		0.91	0.72–1.15	0.434
	Q3	0.99	0.77–1.28	0.958		1.05	0.82–1.34	0.716		1.03	0.79–1.33	0.848
	Q4	0.91	0.65–1.29	0.603		0.89	0.64–1.24	0.484		0.94	0.65–1.35	0.735
No	Q1	1.00 (reference)				1.00 (reference)				1.00 (reference)		
	Q2	0.98	0.90–1.06	0.608		0.97	0.89–1.05	0.443		0.99	0.91–1.07	0.773
	Q3	0.97	0.88–1.06	0.494		0.96	0.88–1.05	0.420		0.96	0.88–1.06	0.427
	Q4	0.90	0.80–1.02	0.096		0.87	0.77–0.98	0.020		0.90	0.79–1.02	0.093

*Multivariate Cox regression model was adjusted for entry age, BMI, pack year smoking, alcohol drinking intensity, total energy, total vegetable intake, total fruit intake, total calcium intake, total folate intake, education, race, marital status, study center, arm and family history

### Combined outcomes of insoluble fiber intake and risk factors for PCa risk

Insoluble fiber intake was significantly associated with the risk of PCa in the subgroup of men of advanced age (> 65 years), former or nonsmokers and men without a family history of PCa. Next, we investigated the combined effects of insoluble fiber intake and age, smoking status and family history on the risk of PCa. As shown in Table 4, we treated men aged > 65 years and with low insoluble fiber intake as a reference group. The aHRs were 0.81 (95% CI, 0.69–0.94) for those aged > 65 years with high insoluble fiber intake, 0.73 (95% CI, 0.66–0.81) for those aged ≤ 65 years with low insoluble fiber intake, and 0.62 (95% CI, 0.54–0.72) for those aged ≤ 65 years with high insoluble fiber intake. Similar results were observed for the joint outcomes of family history and insoluble fiber intake. The aHRs decreased from 1.11 to 0.82 and 0.71 for those with a family history of PCa with high insoluble fiber intake (95% CI, 0.89–1.38), those without a family history of PCa and with low insoluble fiber intake (95% CI, 0.71–0.95), and those without family history of PCa and with high insoluble fiber intake (95% CI, 0.60–0.83), respectively. However, no significant joint outcomes of smoking status or insoluble fiber intake were observed.

**Table 4:** Combined effects of insoluble fiber intake and other risk factors on PCa risk

Variables	Insoluble fiber intake^*^	Controls	PCa cases	aHR^†^	95% CI	*P*
Age						
> 65	Low	3,663	591	1.00 (reference)		
> 65	High	3,481	538	0.81	0.69–0.94	6.41E-03
≤65	Low	8,365	987	0.73	0.66–0.81	3.98E-09
≤65	High	8,537	1,021	0.62	0.54–0.72	2.67E-10
Smoke status						
Current	Low	1,767	177	1.00 (reference)		
Current	High	860	108	1.07	0.82–1.40	0.599
No/Former	Low	10,010	1,376	1.17	0.99–1.37	0.062
No/Former	High	10,935	1,418	0.96	0.79–1.15	0.639
Family history						
Yes	Low	805	163	1.00 (reference)		
Yes	High	817	173	1.11	0.89–1.38	0.350
No	Low	10,639	1,358	0.82	0.71–0.95	8.56E-03
No	High	10,738	1,326	0.71	0.60–0.83	3.49E-05

^*^ Low level: the lowest quartile of insoluble fiber intake (Q1); High level: the highest quartile of insoluble fiber intake (Q4)

^†^ Multivariate Cox regression model was adjusted for entry age, BMI, pack year smoking, alcohol drinking intensity, total energy, total vegetable intake, total fruit intake, total calcium intake, total folate intake, education, race, marital status, study center, arm and family history

### Fiber intake and the prognosis of PCa patients

The relationship between fiber intake and PCa prognosis is summarized in Table 5. The results indicate that total, insoluble and soluble fiber intake are not significantly related to the prognosis of PCa patients (all *P* > 0.05). Compared with patients in the lowest quartile of total, insoluble and soluble fiber intake, patients in the highest quartile of fiber intake experienced no significant protective effect on PCa prognosis. The aHRs of the highest vs. lowest quartile of fiber intake were 1.02 (0.90–1.15, *P* = 0.785) for total fiber, 0.97 (0.87–1.09, *P* = 0.627) for insoluble fiber and 0.95 (0.84–1.08, *P* = 0.433) for soluble fiber.

**Table 5:** Fiber intake and the mortality of PCa in PLCO cohort

Nutrients	Dead	Alive	aHR^*^	95% CI	*P*
Total fiber (g/day)					
Q1 (1.42–12.93)	208	1,399	1.00 (reference)		
Q2 (12.94–17.75)	184	1,418	0.98	0.91–1.06	0.616
Q3 (17.76–23.64)	208	1,394	1.02	0.93–1.11	0.716
Q4 (23.65–88.79)	182	1,421	1.02	0.90–1.15	0.771
Insoluble fiber (g/day)					
Q1 (0.89–8.40)	211	1,399	1.00 (reference)		
Q2 (8.41–11.63)	194	1,409	0.95	0.88–1.03	0.211
Q3 (11.64–15.59)	194	1,408	0.98	0.90–1.06	0.570
Q4 (15.60–57.39)	183	1,416	0.97	0.87–1.09	0.640
Soluble fiber (g/day)					
Q1 (0.51–4.33)	217	1,395	1.00 (reference)		
Q2 (4.34–5.91)	179	1,422	0.95	0.88–1.02	0.173
Q3 (5.92–7.86)	196	1,404	1.00	0.91–1.09	0.958
Q4 (7.87–30.71)	190	1,411	0.95	0.84–1.08	0.438

^*^ Multivariate Cox regression model was adjusted for entry age, BMI, pack year smoking, alcohol drinking intensity, total energy, total vegetable intake, total fruit intake, total calcium intake, total folate intake, education, race, marital status, study center, arm, family history, PSA (prostate specific antigen) and Gleason, clinical stage and PCa histopathologic type

## Discussion

In this study, we found that total fiber, insoluble fiber and soluble fiber all played a protective role against the risk of PCa. Insoluble fiber intake was inversely associated with PCa risk. Nevertheless, total fiber and soluble fiber intake showed no association with mortality in PCa patients. Further subgroup analysis revealed that insoluble fiber intake was associated with decreased PCa risk among patients with the following characteristics: age > 65 years, nonsmoker and former-smoker status, education status ≤ high school, non-Hispanic white ethnicity, or no family history of PCa. In addition, insoluble fiber intake was significantly associated with PCa risk in combination with other factors, including age and family history.

Recently, an increasing number of studies have been performed to investigate the relationship between fiber intake and disease risk. It has been reported that a higher intake of fiber is significantly associated with a decreased risk of peripheral artery disease [26], breast cancer [27], head and neck cancer [28] and colorectal cancer [29, 30]. However, the role of fiber intake in PCa risk remains controversial. According to compliance with the 2018 nutrition-based guidelines of the WCRF/AICR cancer prevention recommendations and prostate cancer, fiber intake has no relationship with PCa risk [31]. A large cohort study from Europe (*n* = 142,590) also demonstrated that dietary fiber intake was not significantly related to the risk of PCa [24]. However, another prospective study of 43,435 men in Japan revealed that insoluble fiber intake but not total or soluble fiber intake was associated with a decreased risk of PCa [23]. Our study also showed that only individuals with an insoluble fiber intake higher than 15.7 g/day (Q4) had a markedly lower risk of PCa, which is consistent with the above results coming from Japan.

Although epidemiological evidence shows a relationship between insoluble fiber intake and PCa risk, the underlying mechanisms remain largely unknown. There are several possible underlying mechanisms. Some studies have demonstrated that dietary fiber improves insulin sensitivity and improves insulin-like growth factor (IGF) dysfunction [32, 33]. Notably, in vitro evidence has shown that insulin resistance and hyperinsulinemia contribute to a high risk of PCa by altering the biological function of IGF-1 or IGF-2 [34, 35]. Additionally, insoluble fiber can be fermented to produce short-chain fatty acids (SCFAs), which play important roles in biological processes including chemotaxis, immune cell immigration, and programmed cell death [36]. Previous evidence has suggested that SCFAs are beneficial for host immunity and metabolism in various organs, such as the digestive system and prostate [36–38]. For instance, butyrate, a type of SCFA, is metabolized from insoluble fiber in the colon. It has been reported to have anti-inflammatory effects [37], and previous studies have indicated that chronic inflammation is involved in the development of PCa [39]. Recent studies have demonstrated that an imbalance in the gut microbiota leads to tumorigenesis in extraintestinal organs, such as the prostate and lung [40, 41]. Insoluble fibers mainly include cellulose, lignin, and hemicellulose, which reduce intestinal transit time and promote regularity of the digestive system. This may provide an excellent environment for the growth of the intestinal flora, promote internal microbiota balance and activate the immune system [42].

The subgroup analysis results of our study indicate that compared with participants who have a insoluble fiber intake in the lowest quartile (Q1), an intake in the highest quartile (Q4) and the following characteristics are significantly associated with decreased PCa risk: male sex, age > 65 years, nonsmoker or former-smoker status, education level of less than high school, non-Hispanic white ethnicity and no family history of PCa. Intriguingly, among current smokers, higher insoluble fiber intake is related to an increased risk of PCa (aHR = 1.49). This is an interesting phenomenon that should be analyzed with a larger sample size as well as the study of underlying mechanisms in the future. In addition, the majority of the study subjects (89%) were white and non-Hispanic, and the number of individuals of other races was relatively small, which may be the reason that no significant associations were observed in the other race groups. Next, we investigated the combined outcome of insoluble fiber intake and other factors, such as age, smoking status and family history of PCa. We treated the high-risk group (elderly individuals and those with lower insoluble fiber intake) as the reference group, and the protective effects gradually became stronger for individuals with higher insoluble fiber intake (aHR = 0.81) or aged ≤ 65 years (aHR = 0.73) alone than for those with both factors (aHR = 0.62). Similar combined outcomes of insoluble fiber intake and family history were also observed. In addition, smoking status had no remarkable combined effect with insoluble fiber intake on PCa risk. These results suggest that insoluble fiber, in addition to its own features, may enhance the protective effect of younger age or a lack of family history of PCa.

Although no protective effect of dietary intake on prostate cancer mortality was found in our study, this does not mean that prostate cancer patients do not need an adequate dietary fiber intake. Daily intake of dietary fiber can ensure the healthy functioning of individuals and be of benefit to their quality of life [42]. Many fiber-rich foods contain other nutrients in addition to dietary fiber, such as phytochemicals (e.g., lycopene and carotenoids), that also have a beneficial effect on the health of PCa patients [43]. Thus, additional studies with larger sample sizes and longer follow-up times are needed.

Our study has some strengths. First, the PLCO trial cohort was large and recruited from different research centers across the USA, making these results highly representative and reliable. Second, many potential confounders were included in the multivariate Cox regression analysis to avoid confounding bias. In addition, we explored not only the association between fiber intake and PCa incidence alone but also its potential combined relationship with other risk factors. Some limitations should be acknowledged in the present study. The outcome was overall PCa, and we did not consider the subtypes of PCa, such as localized cancer and advanced cancer. Another limitation was that smoking status, fiber intake dose, drinking status, height and weight, and education were self-reported and therefore subject to inaccuracy. Moreover, further investigations of the mechanisms of insoluble fiber intake alone and of the combined effects on PCa risk are needed.

## Conclusion

We found that total, insoluble, and soluble dietary fiber all had a protective effect on prostate cancer risk. Among them, insoluble fiber showed a stronger association with PCa risk. Moreover, several factors, such as age, education, smoking history, family history, and race, were significantly involved in reducing the risk of PCa with insoluble fiber. However, further studies are needed to elucidate the underlying mechanisms and determine the specific fiber components associated with these benefits in various populations.

### Electronic supplementary material

Below is the link to the electronic supplementary material.

Supplementary Material 1

Supplementary Material 2

Supplementary Material 3

Supplementary Material 4

## Data Availability

The datasets generated and/or analyzed during the current study are not publicly available due to confidentiality reasons. If necessary, Data can be made available upon reasonable request from the US National Cancer Institute PLCO Cancer Screening Trial Team.
