# Supplementary material for: Higher insoluble fiber intake is associated with a lower risk of prostate cancer: results from the PLCO cohort
Source: BMC Public Health. 2024 Jan 19;24:234. doi: 10.1186/s12889-024-17768-8 (PMC10799495; doi:10.1186/s12889-024-17768-8)
Supplement: Supplementary file 1 — Supplementary Material 1 [file 12889_2024_17768_MOESM1_ESM.docx]

| Sup Table 1. The distribution of smoking status in PLCO cohort | | | | | |  |
| --- | --- | --- | --- | --- | --- | --- |
|  | Controls | |  | Cases | | *P*^*^ |
|  | No. of controls | % |  | No. of cases | % |  |
| Smoking Status |  |  |  |  |  | < 0.001 |
| Never | 17,334 | 36.91 |  | 2,619 | 41.56 |  |
| Current | 4,912 | 10.46 |  | 523 | 8.30 |  |
| Former | 24,718 | 52.63 |  | 3,160 | 50.14 |  |
| Total | 46964 |  |  | 6302 |  |  |
| ^*^ From chi-square test. | | | | | | |
