# Supplementary material for: Higher insoluble fiber intake is associated with a lower risk of prostate cancer: results from the PLCO cohort
Source: BMC Public Health. 2024 Jan 19;24:234. doi: 10.1186/s12889-024-17768-8 (PMC10799495; doi:10.1186/s12889-024-17768-8)
Supplement: Supplementary file 2 — Supplementary Material 2 [file 12889_2024_17768_MOESM2_ESM.docx]

| Sup Table 2. The distribution of stage and histopathologic of prostate cancer cases | | |
| --- | --- | --- |
|  | Cases | |
|  | No. of cases | % |
| Stage |  |  |
| I | 23 | 0.36 |
| II | 6,140 | 95.73 |
| II | 87 | 1.36 |
| IV | 119 | 1.86 |
| Not available | 45 | 0.70 |
| Histological Type |  |  |
| Adenocarcinoma NOS | 6,034 | 94.08 |
| Adenocarcinoma Acinar | 355 | 5.53 |
| Adenocarcinoma Mucinous | 8 | 0.12 |
| Adenocarcinoma Ductal | 3 | 0.05 |
| Small Cell Carcinoma | 2 | 0.03 |
| Carcinoma NOS | 7 | 0.11 |
| Not available | 5 | 0.08 |

Prostate Stage based on Clinical TNM
