# Supplementary material for: Higher insoluble fiber intake is associated with a lower risk of prostate cancer: results from the PLCO cohort
Source: BMC Public Health. 2024 Jan 19;24:234. doi: 10.1186/s12889-024-17768-8 (PMC10799495; doi:10.1186/s12889-024-17768-8)
Supplement: Supplementary file 3 — Supplementary Material 3 [file 12889_2024_17768_MOESM3_ESM.docx]

Sup Table 3. Association between fiber intake and the risk of PCa after 3 years

| Nutrients | aHR*^*^* | 95% CI | *P* |
| --- | --- | --- | --- |
| Total fiber (g/day) |  |  |  |
| Q1 (0.74–12.84) | 1.00 (reference) | |  |
| Q2 (12.85–17.70) | 0.93 | 0.85–1.01 | 0.094 |
| Q3 (17.71–23.74) | 0.95 | 0.86–1.05 | 0.315 |
| Q4 (23.75–97.82) | 0.90 | 0.78–1.03 | 0.113 |
| Insoluble fiber (g/day) |  |  |  |
| Q1 (0.42–8.34) | 1.00 (reference) | |  |
| Q2 (8.35–11.60) | 0.94 | 0.86–1.03 | 0.162 |
| Q3 (11.61–15.69) | 0.96 | 0.87–1.06 | 0.399 |
| Q4 (15.70–65.67) | 0.84 | 0.74–0.96 | 0.009 |
| Soluble fiber (g/day) |  |  |  |
| Q1 (0.31–4.31) | 1.00 (reference) | |  |
| Q2 (4.32–5.90) | 0.93 | 0.86–1.02 | 0.137 |
| Q3 (5.91–7.91) | 0.97 | 0.87–1.07 | 0.519 |
| Q4 (7.92–36.66) | 0.89 | 0.77–1.03 | 0.120 |

^*^Multivariate Cox regression model was adjusted for entry age, BMI, pack year smoking, alcohol drinking intensity, total energy, total vegetable intake, total fruit intake, total calcium intake, total folate intake, education, race, marital status, study center, arm and family history.
