# Supplementary material for: Higher insoluble fiber intake is associated with a lower risk of prostate cancer: results from the PLCO cohort
Source: BMC Public Health. 2024 Jan 19;24:234. doi: 10.1186/s12889-024-17768-8 (PMC10799495; doi:10.1186/s12889-024-17768-8)
Supplement: Supplementary file 4 — Supplementary Material 4 [file 12889_2024_17768_MOESM4_ESM.pdf]

AJE®

Editing Certificate

This document certifies that the manuscript

Higher insoluble fiber intake is associated with lower risk of prostate cancer: results from the PLCO cohort

prepared by the authors

Shen Yang

was edited for proper English language, grammar, punctuation, spelling, and overall style by one or more of the highly qualified native English speaking editors at AJE.

This certificate was issued on **December 30, 2023** and may be verified on the AJE website using the verification code **F73D-758E-453C-CD96-4BCP**.

AJE® EDITING CERTIFIED

Neither the research content nor the authors' intentions were altered in any way during the editing process. Documents receiving this certification should be English-ready for publication; however, the author has the ability to accept or reject our suggestions and changes. To verify the final AJE edited version, please visit our verification page at aje.com/certificate. If you have any questions or concerns about this edited document, please contact AJE at support@aje.com.

AJE provides a range of editing, translation, and manuscript services for researchers and publishers around the world.
For more information about our company, services, and partner discounts, please visit aje.com.
